# Association Between Frequent PVCs and Myocardial Strain in Children with Structurally Normal Hearts

**DOI:** 10.3390/children13050667

**Published:** 2026-05-11

**Authors:** Hilmi Onur Kabukçu, Pelin Köşger, Ayşe Sülü, Birsen Uçar

**Affiliations:** 1 Department of Pediatrics, Private Kastamonu Anatolia Hospital, 37200 Kastamonu, Turkey; 2 Department of Pediatric Cardiology, Faculty of Medicine, Eskişehir Osmangazi University, 26040 Eskişehir, Turkey; pkosger@ogu.edu.tr (P.K.); bucar@ogu.edu.tr (B.U.); 3 Department of Pediatric Cardiology, Faculty of Medicine, Gaziantep University, 27410 Gaziantep, Turkey; suluayse@windowslive.com

**Keywords:** myocardial function, pediatric, premature ventricular contraction, strain

## Abstract

**Highlights:**

**What are the main findings?**

• Children with frequent PVCs showed impaired myocardial strain despite preserved ejection fraction.
• PVC burden was positively correlated with worsening global longitudinal strain values.

**What are the implications of the main findings?**

• Myocardial strain analysis may detect early subclinical dysfunction in pediatric patients with frequent PVCs.
• Strain imaging could be useful in guiding clinical follow-up and management decisions in this population.

**Abstract:**

Background/Objectives: While premature ventricular contraction (PVC) burden is clearly linked to cardiomyopathy in adults, its association with ventricular dysfunction in children remains less well established. This study investigated the myocardial effects of a PVC burden greater than 5% and its potential predictive factors in a pediatric population. Methods: The study enrolled 23 children aged 5–18 years with a PVC burden >5% on 24 h Holter monitoring, who had no chronic systemic illness, congenital or acquired heart disease, or known family history of cardiomyopathy or sudden cardiac death, along with 33 age-matched healthy controls. Data on demographic characteristics, anthropometric measures, clinical findings, and laboratory results were obtained. Twelve-lead electrocardiography, exercise testing using the Bruce protocol, 24 h Holter monitoring, and echocardiographic assessments—including conventional, tissue Doppler, and both segmental and global strain analyses—were performed and compared between the patient and control groups. Results: In the patient group, left ventricular isovolumetric relaxation time (IVRT) was prolonged, and the myocardial performance index (MPI) was higher compared with the controls (*p* < 0.001, *p* = 0.004). Longitudinal strain analysis revealed a significant reduction in global longitudinal strain (GLS) (*p* = 0.035). In addition, significantly lower segmental longitudinal strain values were observed in the basal anterolateral, basal inferolateral, and basal anterior segments, as well as a reduction in apical two-chamber GLS (*p* = 0.002, *p* = 0.002, *p* = 0.003, and *p* = 0.014, respectively). Circumferential strain was also significantly reduced in the basal anteroseptal, basal anterior, basal inferolateral, and mid anteroseptal segments, as well as in the basal, mid, and global averages (*p* = 0.003, *p* = 0.003, *p* = 0.026, *p* = 0.003, *p* = 0.006, *p* = 0.022, and *p* = 0.017, respectively). PVC burden on Holter monitoring was positively correlated with the global strain values, indicating less negative strain with increasing PVC burden. Conclusions: In children with structurally normal hearts and a PVC burden exceeding 5%, TDI and strain imaging revealed subtle alterations in diastolic function and myocardial deformation despite preserved ejection fraction. These findings suggest that frequent PVCs may be associated with early myocardial alterations and highlight the potential utility of advanced echocardiographic techniques in this population.

## 1. Introduction

Premature ventricular contractions (PVCs) are relatively common in otherwise healthy children and adolescents, with a prevalence of 18–50% reported in ambulatory monitoring studies. During childhood, PVCs are most often asymptomatic and identified incidentally. They typically decrease with exercise, may resolve spontaneously over time, and only rarely require intervention. However, in some cases, PVCs can be an early indicator of underlying myocardial disease or channelopathy [1].

In adults, a clear association has been demonstrated between PVC burden and the development of cardiomyopathy. Left ventricular systolic dysfunction observed in patients with a PVC burden greater than 24% has been shown to improve following catheter ablation [2]. Facchini et al. [3] compared 57 adults with at least 30 PVCs per hour and no structural heart disease to 32 healthy controls, and found that patients with ventricular ectopy had a significantly larger left ventricular end-diastolic diameter (50 ± 6 mm vs. 47 ± 3 mm). In children, however, the relationship between PVC burden and ventricular dysfunction remains less well defined. Abadir et al. [4] reported no correlation between PVC burden >10% and LVEF in a cohort of 47 patients younger than 18 years. Conversely, Sun et al. [5] demonstrated that children with frequent PVCs (>10 PVCs/min) and a short coupling interval (RR’/RR ≤ 0.6) had a significantly reduced mean LVEF and cardiac index. Moreover, additional factors such as bigeminy, trigeminy, quadrigeminy, QRS morphology of the PVC, coupling interval, and the presence or absence of symptoms have also been implicated as relevant determinants [6]. These discrepancies highlight the need for further studies specifically addressing the relationship between PVCs and myocardial function in pediatric patients, as well as the role of associated factors, particularly through sensitive modalities such as speckle-tracking echocardiography.

In this study, we aimed to evaluate myocardial function in children without structural heart disease who had a PVC burden greater than 5%, and to investigate whether left ventricular function was associated with PVC burden, morphology, site of origin, coupling interval, QRS duration, and exercise test response.

## 2. Materials and Methods

Medical records of 37 patients aged 6–18 years who presented to the Pediatric Cardiology Department of Eskişehir Osmangazi University Medical School between 1 January 2020 and 1 August 2021, with a PVC burden greater than 5% on Holter ECG recordings were reviewed retrospectively. Six patients with structural heart disease, prior cardiac surgery, cardiomyopathy, long QT syndrome, antiarrhythmic medication use, history of leukemia, along with eight patients who were lost to follow-up during the COVID-19 pandemic were excluded. Clinical data from 23 patients and 33 healthy children without PVCs on 24 h Holter monitoring were compared. Demographic and clinical characteristics such as sex, age, weight, height, body mass index, symptoms and laboratory test parameters were recorded.

Ventricular origin of PVCs, transition zones, QRS duration, coupling interval, heart rate and cQT duration were identified using resting 12-lead ECG. PVCs were categorized according to 12-lead ECG morphology, frontal QRS axis, and precordial transition (1). PVCs with LBBB morphology, inferior axis, and a transition around V3 were classified as RVOT origin. PVCs with RBBB morphology, inferior axis, and a transition around V3 were classified as LVOT origin. PVCs with LBBB morphology, superior axis, and a transition around V3 were classified as non-outflow tract PVCs, consistent with an inferior/apical RV origin based on ECG criteria.

A 24 h Holter ECG monitoring was performed using a three-channel recorder (DMS 300-7 HolterReader; DMS, Stateline, NV, USA). Recordings were analyzed offline with Holter LX Analysis software QLAB 10, Philips Medical Systems, Andover, MA, USA (DM Software Inc., Stateline, NV, USA). The PVC burden (percentage of PVCs among total beats), PVC morphology, heart rate dependency (suppression or increase with higher heart rates), and the presence of complex ventricular ectopy—such as bigeminy, trigeminy, couplets, nonsustained runs, or ventricular tachycardia (VT)—were evaluated from Holter recordings. Frequent PVCs were defined as a burden >5% over 24 h.

### Echocardiographic Assessment and Strain Analysis

Echocardiographic studies were performed using a high-quality echocardiography system (Philips EPIQ CVx, Philips Healthcare, Andover, MA, USA). Routine transthoracic echocardiography was conducted in accordance with the 2014 Appropriate Use Criteria for Initial Transthoracic Echocardiography in Outpatient Pediatric Cardiology (7). Left ventricular (LV) ejection fraction was assessed using the Teichholz formula. LV systolic dysfunction was defined as an LV ejection fraction <55% or fractional shortening ≤28% (8). Left ventricular ejection fraction (LVEF), shortening fraction (SF), LV end-diastolic diameter (LVEDd), right ventricular end-diastolic diameter (RVEDd), tissue Doppler imaging parameters, TDI-derived myocardial performance index (MPI), and two-dimensional speckle-tracking echocardiography (STE) measurements were obtained during sinus rhythm in clinically stable patients at rest. Blood pressure and heart rate were within age-appropriate ranges at the time of imaging, and patients with acute illness or hemodynamic instability were not included.

**Figure 1:**
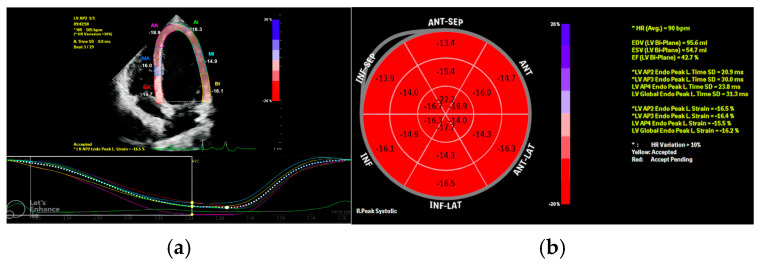
Representative two-dimensional speckle-tracking echocardiography images obtained from a 15-year-old female patient with a PVC burden of 6%. (**a**) shows the apical long-axis view with segmental longitudinal strain curves. (**b**) demonstrates the corresponding 17-segment bull’s-eye plot, with a global longitudinal strain (GLS) of −16.2%.

For STE analysis, apical four-, three-, and two-chamber views and parasternal short-axis images were acquired using conventional two-dimensional gray-scale echocardiography with a stable ECG recording. Images were obtained at frame rates appropriate for pediatric STE (approximately 60–90 frames/s). Strain analyses were performed offline using the manufacturer’s dedicated software (QLAB10, Philips Medical Systems, Andover, MA, USA). Endocardial borders were manually traced, and the region of interest was automatically generated and adjusted when necessary to include the entire myocardial thickness. Tracking quality was visually verified for each segment throughout the cardiac cycle. Segments with suboptimal image quality or inadequate tracking (drop-out or mistracking) were re-traced, and if reliable tracking could not be achieved, they were excluded from segmental analysis. Peak systolic longitudinal strain values were calculated using a 17-segment LV model. A bull’s-eye plot summarizing all three apical planes was used to derive global longitudinal strain (GLS) (Figure 1), and global circumferential strain (GCS) was measured from the parasternal short-axis view. All strain analyses were performed offline by the same experienced pediatric cardiologist to minimize inter-observer variability.

The study was granted ethical approval by the local Clinical Research Ethics Committee of Eskişehir Osmangazi University on 27 June 2019. The study was performed in accordance with the principles outlined in the Declaration of Helsinki. Normality was assessed using the Shapiro–Walk normality test and continuous variables were described using mean ± standard deviation (SD) or median (25–75%) as appropriate. Categorical variables were expressed as absolute numbers and percentages. T-test was employed for comparisons involving normally distributed data whereas non-normally distributed data were compared using the Mann–Whitney U test. For categorical variables, a chi-square test was performed. The statistical significance level was set as *p* < 0.05. IBM SPSS Statistics for Windows, Version 21.0 (IBM Corp., Armonk, NY, USA) (SPSS Inc., Chicago, IL, USA).

## 3. Results

The mean age of the patients was 164 ± 38 months and there was no significant difference between the groups regarding age, weight, height, body mass index, sex and laboratory parameters (*p* > 0.05). Palpitation was the most common presenting symptom followed by chest pain as the second most frequently reported complaint (Table 1).

The mean PVC frequency was 11,879 beats/24 h (5655–39,073 beats) with 17 patients having a 5–10% and six patients having a greater than 10% PVC burden. According to 12-lead ECG evaluation, PVCs originated from the right ventricular outflow tract with a left bundle branch block morphology and an inferior axis in 14 patients, and from a non-outflow tract right ventricular origin with a left bundle branch block morphology and a superior axis in two patients. All patients with right ventricular PVCs demonstrated a late precordial transition (V3–V4 or later). The remaining seven patients were classified as having a left ventricular origin late precordial transition; among these, six exhibited a right bundle branch block morphology with an inferior axis whereas one patient demonstrated a superior axis (Table 2).

Corrected QT interval and heart rate were similar between the groups. The 24 h Holter monitoring of one patient who had a PCV burden greater than 10% revealed 136 episodes of ventricular run, each consisting of three consecutive beats. No sustained ventricular tachycardia was observed. During the treadmill stress test, PVCs disappeared in 13 patients, decreased in four patients, showed no changes in five patients and increased in one patient. Ventricular tachycardia was not observed in any patient (Table 2).

Conventional 2D and M-mode echocardiographic parameters were comparable between patients and controls; however, tissue Doppler imaging revealed subtle functional differences, with a significantly prolonged LV TDI-derived isovolumic relaxation time (IVRT) and an increased TDI-derived myocardial performance index (MPI) in patients (both *p* < 0.05) (Table 3).

In the patient group, longitudinal strain analysis revealed significantly lower global and regional strain in the basal anterolateral, basal inferolateral, basal anterior and apical two chamber segments compared to the healthy group. Similarly circumferential strain analysis demonstrated significantly more depressed basal anterolateral, basal anterior, basal inferoseptal, mid-anteroseptal and global circumferential strain values compared to healthy subjects (*p* = 0.002, *p* = 0.002, *p* = 0.003, *p* = 0.030, *p* = 0.003, *p* = 0.003, *p* = 0.026, *p* = 0.003, *p* = 0.006, *p* = 0.022, *p* = 0.017, respectively) (Table 4 and Table 5).

There was a significant correlation between the PVC burden and global longitudinal and circumferential strain values (r = 0.337, *p* = 0.012; r = 0.290, *p* = 0.035, respectively).

After adjustment for age, sex, heart rate, hemoglobin level, systolic blood pressure, and LV end-diastolic dimension (M-mode LVIDd), GLS remained significantly correlated with ventricular ectopic burden (r = 0.323, *p* = 0.024).

## 4. Discussion

In our cohort of children and adolescents with a PVC burden >5%, conventional echocardiography did not reveal overt structural abnormalities or clear systolic dysfunction. However, speckle-tracking echocardiography identified both segmental and global differences in longitudinal and circumferential strain that correlated with PVC burden, despite preserved ejection fraction. These findings suggest that frequent PVCs may be associated with subtle, subclinical alterations in myocardial mechanics that can be underestimated by routine echocardiographic measures. This supports the concept that early myocardial involvement in structurally normal pediatric hearts may initially manifest as altered myocardial deformation rather than changes in chamber dimensions or global systolic indices. Notably, the concurrent prolongation of IVRT and elevation of MPI, while other Doppler parameters remained normal, adds a complementary mechanics-based signal that the ventricular contraction–relaxation cycle may be mildly affected before standard echocardiographic abnormalities emerge.

In adults, multiple studies have consistently demonstrated that a higher PVC burden is associated with left ventricular dilatation and systolic dysfunction, abnormalities that may be reversible following catheter ablation [2,3,7]. In contrast, data in the pediatric population remain less consistent. While some studies have failed to show a significant relationship between PVC burden and ventricular function, others have reported systolic impairment in children with frequent PVCs [8,9]. A recent comprehensive meta-analysis including 17 studies and 1701 pediatric patients reported a 1.7% prevalence of PVC-related cardiomyopathy [10] and consistently underscored a strong association between PVC burden and adverse alterations in myocardial systolic function, with PVC-induced cardiomyopathy being linked to a longer QRS duration and shorter coupling intervals [11,12]. Similar findings were reported by Chen et al. [13], who demonstrated that pediatric patients with a PVC burden >20% had significantly larger left ventricular dimensions and reduced systolic function, with normalization after ablation in affected cases.

In the present study, left ventricular ejection fraction remained preserved and comparable to that of healthy peers, whereas modest but statistically significant differences in longitudinal and circumferential strain parameters were observed. Importantly, mean strain values in both groups remained within the generally accepted normal range, and therefore should not be interpreted as evidence of overt myocardial dysfunction.

Rather, the relatively reduced deformation parameters and their association with PVC burden may reflect subtle, subclinical myocardial alterations. Relative differences within the normal range may still carry pathophysiological relevance, particularly in conditions where early myocardial involvement is expected. In this context, myocardial strain analysis may offer incremental value over conventional echocardiographic parameters by enabling the detection of early functional changes before the development of overt systolic impairment [11,14].

As in other pediatric series, most idiopathic PVCs in our cohort exhibited an LBBB morphology, indicating a predominantly right ventricular origin. An LBBB-type activation sequence is known to induce ventricular mechanical dyssynchrony, and chronic dyssynchronous activation, well illustrated in right ventricular apical pacing models, can reduce mechanical efficiency and promote subtle functional deterioration over time [15,16]. In line with this concept, the observed reductions in global longitudinal and circumferential strain, despite preserved ejection fraction, may reflect a mild dyssynchrony-related mechanical effect rather than overt systolic dysfunction. In our cohort, however, comprehensive echocardiographic evaluation demonstrated structurally normal hearts without additional repolarization abnormalities, supporting the diagnosis of idiopathic PVCs. Cardiac magnetic resonance imaging was not performed systematically because of the absence of clinical indications such as ventricular dysfunction, abnormal baseline ECG findings, or a positive family history; therefore, a concealed cardiomyopathic substrate cannot be entirely excluded in a subset of patients. Overall, these findings underscore the importance of periodic reassessment of PVC burden and serial evaluation of ventricular function and myocardial mechanics, particularly in children with predominant LBBB morphology PVCs and a superior axis [6].

Exercise testing provided an additional risk stratification tool in our cohort. In most patients, PVCs decreased or disappeared with increasing workload, and exercise capacity was comparable to healthy peers, a response generally considered reassuring in children with structurally normal hearts.

Conversely, exercise-induced augmentation of ectopy, polymorphic PVCs, or ventricular tachycardia during exercise or early recovery are viewed as higher-risk features that would prompt closer surveillance and further evaluation [17,18]. In our cohort, the predominantly favorable exercise response supports the overall low-risk clinical profile of the study population and is consistent with the absence of overt systolic dysfunction despite subtle alterations in myocardial deformation parameters.

These findings suggest that, in patients without high-risk exercise characteristics, mildly reduced strain values within the normal range may not translate into immediate clinical risk but may instead represent early, subclinical changes. Nevertheless, because adverse outcomes may rarely occur even in the absence of exercise-induced ectopy, continued longitudinal follow-up remains appropriate.

This study has certain limitations. Cardiac MRI was not performed systematically in this retrospective cohort; therefore, the presence of a subtle or early cardiomyopathic substrate cannot be entirely excluded. In addition, although patients were included based on a ≥5% PVC burden with the intention of performing further subgroup analyses (e.g., 5–10% vs. >10%), the limited number of patients—particularly in the higher-burden subgroup—precluded meaningful statistical comparisons. Given the relatively small sample size and the single-center, cross-sectional nature of the study, larger, preferably prospective and multicenter studies are needed to validate and extend our findings.

In summary, in children with frequent PVCs, conventional echocardiographic indices, including LVEF, remained within normal limits, whereas speckle-tracking echocardiography revealed subtle left ventricular abnormalities consistent with early alterations in myocardial mechanics. As one of the few studies to apply STE in this clinical context, our findings suggest that strain imaging provides incremental value beyond standard echocardiography and may be particularly useful for the longitudinal follow-up of children with a PVC burden >5%, allowing earlier recognition of functional changes and supporting a more individualized management approach.

However, given the cross-sectional design, relatively small sample size, and the lack of systematic cardiac MRI evaluation, these findings should be interpreted with caution. It remains unclear whether these abnormalities represent early stages of a progressive process or benign variations, warranting confirmation in larger prospective multicenter studies incorporating advanced imaging modalities.

## Figures and Tables

**Table 1:** Baseline demographic, clinical and laboratory characteristics of the patient and control groups.

Variable	Patient Group (n = 23)	Control Group (n = 33)	*p* Value
Demographic data			
Age, months	164.1 ± 38.8	148.8 ± 43.3	0.181
Weight, kg	53.1 ± 16.7	44.8 ± 16.6	0.076
Height, cm	158.1 ± 16.1	149.0 ± 19.5	0.071
BMI, kg/m^2^	20.7 ± 3.7	19.7 ± 3.5	0.290
Clinical data			
Sex, female	10 (43.5)	23 (69.7)	0.092
Sex, male	13 (56.5)	10 (30.3)	
Family history, positive	-	-	
Presenting symptom			
None	5 (21.7)	7 (21.2)	0.199
Palpitation	8 (34.8)	6 (18.2)	
Dizziness	2 (8.7)	0 (0)	
Chest pain	6 (26.1)	18 (54.5)	
Syncope	0 (0)	0 (0)	
Easy fatigability	2 (8.7)	2 (6.1)	
Blood pressure			
DBP, mmHg	65.1 ± 9.6	61.1 ± 9.6	0.135
SBP, mmHg	113.2 ± 13.9	115.2 ± 10.0	0.569
Laboratory values			
Hb, g/dL	14.0 (13.0–14.8)	13.7 (12.8–14.3)	0.641
TSH, µIU/mL	2.56 (2.18–3.12)	2.74 (1.62–3.38)	0.623
Free T4, ng/dL	1.28 (1.13–1.35)	1.26 (1.19–1.37)	0.484

Data are presented as mean ± standard deviation or median (interquartile range), as appropriate. DBP: diastolic blood pressure, Hb: hemoglobin, SBP: systolic blood pressure, TSH: thyroid-stimulating hormone, BMI: body mass index.

**Table 2:** Electrocardiographic, exercise test and Holter monitoring findings in the patient and control groups.

Variable	Patient Group (n = 23)	Control Group (n = 33)	*p* Value
Electrocardiographic findings			
Heart rate (beats/min)	90 ± 13	88 ± 13	0.612
QTc interval (s)	0.41 ± 0.03	0.40 ± 0.02	0.239
QT interval (s)	0.33 (0.32–0.36)	0.33 (0.32–0.36)	0.890
Exercise test			
Exercise duration (min)	10.4 ± 2.6	9.6 ± 2.0	0.219
Maximal heart rate (beats/min)	196 (187–206)	194 (180–201)	0.744
Change in PVC frequency during exercise test, n (%)			
No change	5 (21.7)	NA	NA
Increased	1 (4.3)	NA	NA
Decreased	4 (17.4)	NA	NA
Disappeared	13 (56.5)	NA	NA
Holter monitoring			
Mean heart rate (beats/24 h)	84 ± 12	88 ± 9	0.223
Total beats (beats/24 h)	120,703 ± 17,433	124,847 ± 13,104	0.314
PVC count (beats/24 h)	11,879 (5655–39,073)	NA	NA
PVC morphology, n (%)			
Left bundle branch block	16 (69.6)	NA	NA
Right bundle branch block	7 (30.4)	NA	NA

Data are presented as mean ± standard deviation or median (interquartile range), as appropriate. NA, not applicable, PVC, premature ventricular contraction.

**Table 3:** Conventional and tissue Doppler echocardiographic parameters in the patient and control groups.

Variable	Patient Group (n = 23)	Control Group (n = 33)	*p* Value
LVEDD index, mm/m^2^	31.9 ± 6.8	33.1 ± 6.3	0.488
EF, %	70.4 ± 6.1	69.9 ± 5.6	0.774
FS, %	39.9 ± 4.7	38.8 ± 6.6	0.310
Em, m/s	12.06 ± 1.91	11.87 ± 1.60	0.684
Am, m/s	6.8 (5.5–7.8)	6.2 (5.5–7.0)	0.695
E, m/s	88.2 ± 21.7	81.8 ± 12.3	0.160
A, m/s	57.6 ± 10.5	55.4 ± 11.7	0.609
E/Em	7.36 ± 1.62	6.95 ± 1.29	0.291
E/A	1.62 ± 0.47	1.50 ± 0.26	0.246
IVRT, ms	58 (50–63)	47 (41–51)	<0.001 *
IVCT, ms	48 (42–53)	47 (40–53)	0.719
ET, ms	275.6 ± 22.4	268.7 ± 22.7	0.262
MPI	0.39 ± 0.06	0.34 ± 0.46	0.004 *
MAPSE, mm	14.48 ± 2.37	13.60 ± 1.64	0.109
TAPSE, mm	21.7 ± 3.04	20.6 ± 5.03	0.352
RVEDD index, mm/m^2^	24.5 ± 5.4	24.2 ± 6.9	0.863

LVEDD, left ventricular end-diastolic diameter; RVEDD, right ventricular end-diastolic diameter; EF, ejection fraction; FS, fractional shortening; Em, early diastolic mitral annular velocity; Am, late diastolic mitral annular velocity; E, early transmitral flow velocity; A, late transmitral flow velocity; IVRT, isovolumic relaxation time; IVCT, isovolumic contraction time; ET, ejection time; MPI, myocardial performance index; MAPSE, mitral annular plane systolic excursion; TAPSE, tricuspid annular plane systolic excursion. * *p* < 0.05 indicates statistical significance.

**Table 4:** Segmental and global longitudinal strain values (%) in the patient and control groups.

Segment	Patient Group (n = 23)	Control Group (n = 33)	*p* Value
A4C view			
BIS	−19.6 ± 4.4	−21.1 ± 4.1	0.223
BAL	−15.9 (−21.2 to −14.3)	−21.4 (−24.1 to −19.1)	0.002 *
MIS	−21.1 ± 4.1	−23.1 ± 3.2	0.069
MAL	−17.5 ± 5.6	−19.8 ± 3.6	0.065
APS	−21.6 (−23.8 to −20.7)	−23.7 (−26.1 to −21.7)	0.140
APL	−19.5 (−22.6 to −17.5)	−20.4 (−22.3 to −18.5)	0.947
A3C view			
BAS	−18.1 ± 3.7	−20.3 ± 5.1	0.079
BIL	−19.6 ± 3.3	−24.1 ± 6.9	0.002 *
MAS	−21.0 ± 3.7	−21.2 ± 2.7	0.789
MIL	−19.5 ± 3.4	−21.1 ± 4.2	0.116
APA	−21.2 ± 4.0	−21.7 ± 3.5	0.693
A2C view			
BA	−21.1 (−23.9 to −16.1)	−25.5 (−28.5 to −22.7)	0.003 *
BI	−23.6 ± 5.5	−24.2 ± 6.0	0.704
MA	−20.4 ± 4.4	−22.1 ± 3.0	0.100
MI	−19.5 ± 4.8	−20.4 ± 3.2	0.394
API	−17.4 ± 5.6	−18.5 ± 4.4	0.402
Global values			
A4C total	−20.5 ± 3.2	−21.6 ± 2.8	0.168
A3C total	−20.8 ± 3.3	−21.8± 2.5	0.171
A2C total	−19.9 ± 3.9	−22.1± 2.1	0.014 *
GC total	−20.4 ± 3.06	−21.8± 1.8	0.035

Data are presented as mean ± standard deviation or median (interquartile range), according to the distribution of each variable. A4C, apical four-chamber; A3C, apical three-chamber; A2C, apical two-chamber; APA, apical anterior; API, apical inferior; APL, apical lateral; APS, apical septal; BA, basal anterior; BAL, basal anterolateral; BAS, basal anteroseptal; BI, basal inferior; BIL, basal inferolateral; BIS, basal inferoseptal; MA, mid-anterior; MAL, mid-anterolateral; MAS, mid-anteroseptal; MI, mid-inferior; MIL, mid-inferolateral; MIS, mid-inferoseptal; GC, global longitudinal strain. * *p* < 0.05 indicates statistical significance.

**Table 5:** Basal, mid-ventricular and apical circumferential strain values (%) in the patient and control groups.

Segment	Patient Group (n = 23)	Control Group (n = 33)	*p* Value
Basal (%)			
BAS	−17.1 ± 6.4	−22.4 ± 5.9	0.003 *
BA	−21.1 (−23.1 to −16.1)	−25.5 (−28.5 to −22.7)	0.003 *
BAL	−14.6 (−20.5 to −15.4)	−18.9 (−22.3 to −15.4)	0.395
BIL	−14.3 (−20.5 to −15.9)	−21.1 (−25.7 to −16.3)	0.026 *
BI	−19.2 ± 7.5	−23.7 ± 6.3	0.190
BIS	−14.5 (−17.1 to −13.6)	−14.6 (−18.4 to −11.7)	0.655
Middle (%)			
MAS	−20.4 ± 5.3	−24.8 ± 4.9	0.003 *
MA	−21.6 ± 6.7	−25.2 ± 4.8	0.220
MAL	−20.7 ± 4.9	−23.1 ± 6.3	0.086
MIL	−21.7 ± 5.4	−24.8 ± 5.8	0.058
MI	−21.8 ± 6.2	−24.8 ± 5.1	0.059
MIS	−19.3 ± 4.3	−22.4 ± 4.9	0.220
Apical (%)			
APS	−23.8 ± 9.0	−24.7 ± 6.3	0.648
APA	−25.4 ± 8.3	−27.0 ± 6.0	0.401
APL	−23.4 (−33.2 to −19.2)	−24.9 (−28.1 to −22.7)	0.655
API	−25.5 ± 11.0	−26.0 ± 7.1	0.848
Total circumferential strain (%)			
Basal total	−17.9 (−21.4 to −15.2)	−21.4 (−24.3 to −19.2)	0.006 *
Middle total	−21.4 ± 4.9	−24.5 ± 4.4	0.022 *
Apical total	−24.6 (−31.6 to −20.0)	−25.7 (−26.5 to −22.7)	0.655
Global total	−21.1 ± 4.8	−23.9 ± 3.5	0.017 *

Data are presented as mean ± standard deviation or median (interquartile range), according to the distribution of each variable. BAS, basal anteroseptal; BA, basal anterior; BAL, basal anterolateral; BIL, basal inferolateral; BI, basal inferior; BIS, basal inferoseptal; MAS, mid-anteroseptal; MA, mid-anterior; MAL, mid-anterolateral; MIL, mid-inferolateral; MI, mid-inferior; MIS, mid-inferoseptal; APS, apical septal; APA, apical anterior; APL, apical lateral; API, apical inferior. * *p* < 0.05 indicates statistical significance.

## Data Availability

The data presented in this study are available on request from the corresponding author (Hilmi Onur Kabukçu). The data are not publicly available due to privacy and ethical restrictions.

## Abbreviations

The following abbreviations are used in this manuscript:

PVCs	Premature ventricular contractions
SF	Shortening fraction
LVEF	Left ventricular ejection fraction
LVEDd	Left ventricular end-diastolic diameter
RVEDd	Right ventricular end-diastolic diameter
MPI	Myocardial performance index
IVRT	Left ventricular isovolumetric relaxation time
ECG	Electrocardiography
RVEF	Right ventricular ejection fraction
LBBB	Left bundle branch block
RVOT	Right ventricular outflow tract
LVOT	Left ventricular outflow tract
GLS	Global longitudinal strain
GCS	Global circumferential strain
STE	Speckle-tracking echocardiography

## References

[1] European Society of Cardiology (ESC) (2022). 2022 ESC Guidelines for the Management of Patients with Ventricular Arrhythmias and the Prevention of Sudden Cardiac Death: Developed by the ESC Task Force and Endorsed by the Association for European Paediatric and Congenital Cardiology (AEPC). Eur. Heart J..

[2] Baman T.S., Lange D.C., Ilg K.J., Gupta S.K., Liu T.Y., Alguire C., Armstrong W., Good E., Chugh A., Jongnarangsin K. (2010). Relationship between Burden of Premature Ventricular Complexes and Left Ventricular Function. Heart Rhythm.

[3] Facchini M., Malfatto G., Ciambellotti F., Chianca R., Bragato R., Branzi G., Leonetti G. (1999). Increased Left Ventricular Dimensions in Patients with Frequent Nonsustained Ventricular Arrhythmia and No Evidence of Underlying Heart Disease. J. Cardiovasc. Electrophysiol..

[4] Abadir S., Blanchet C., Fournier A., Mavad V., Shohoudi A., Dahdah N., Hayri P. (2016). Characteristics of Premature Ventricular Contractions and Their Impact on Left Ventricular Function in Healthy Children. Heart Rhythm.

[5] Sun Y., Blom N.A., Yu Y., Ma P., Wang Y., Han X., Swenne C.A., van der Wall E.E. (2003). Effect of Premature Ventricular Contractions on Left Ventricular Function in Asymptomatic Children without Structural Heart Disease: An Echocardiographic Assessment. Int. J. Cardiovasc. Imaging.

[6] Cohen M.B. (2019). Frequent Premature Ventricular Contractions in Healthy Children: When to Ignore and When to Treat?. Curr. Opin. Cardiol..

[7] Takemoto M., Yoshimura H., Ohba Y., Matsumoto Y., Yamamoto U., Mohri M., Yamamoto H., Origuchi H. (2005). Radiofrequency Catheter Ablation of Premature Ventricular Complexes from Right Ventricular Outflow Tract Improves Left Ventricular Dilation and Clinical Status in Patients without Structural Heart Disease. J. Am. Coll. Cardiol..

[8] Kakavand B., Ballard H.O., Disessa T.G. (2010). Frequent Ventricular Premature Beats in Children with a Structurally Normal Heart: A Cause for Reversible Left Ventricular Dysfunction. Pediatr. Cardiol..

[9] Guerrier K., Anderson J.B., Czosek R.J., Mays W.A., Statile C., Knilans T.K., Spar D.S. (2015). Usefulness of Ventricular Premature Complexes in Asymptomatic Patients ≤21 Years as Predictors of Poor Left Ventricular Function. Am. J. Cardiol..

[10] Flore F., Lioncino M., Cicenia M., Garozzo D., Raimondo C., Di Mambro C., Silvetti M.S., Drago F. (2025). Premature Ventricular Contraction-Induced Ventricular Dysfunction in Children without Structural Heart Disease: A Systematic Review and Meta-Analysis. Europace.

[11] Bertels R.A., Harteveld L.M., Filippini L.H., Clur S.A., Blom N.A. (2017). Left Ventricular Dysfunction Is Associated with Frequent Premature Ventricular Complexes and Asymptomatic Ventricular Tachycardia in Children. Europace.

[12] Przybylski R., Meziab O., Gauvreau K., Dionne A., DeWitt E.S., Bezzerides V.J., Abrams D.J. (2024). Premature Ventricular Contractions in Children and Young Adults: Natural History and Clinical Implications. Europace.

[13] Chen B., Li J., Li S., Fang Y., Zhao P. (2020). Risk Factors for Left Ventricular Enlargement in Children with Frequent Premature Ventricular Complexes. Am. J. Cardiol..

[14] Barutçu A., Bekler A., Temiz A., Kırılmaz B., Gazi E., Altun B., Özdemir S., Ulusoy Aksu F. (2016). Assessment of the Effects of Frequent Ventricular Extrasystoles on the Left Ventricle Using Speckle Tracking Echocardiography in Apparently Normal Hearts. Anatol. J. Cardiol..

[15] Littmann L., Symanski J.D. (2000). Hemodynamic Implications of Left Bundle Branch Block. J. Electrocardiol..

[16] Naqvi T.Z., Chao C.-J. (2023). Adverse Impact of Right Ventricular Pacing on Cardiac Function: Prevalence, Prevention, and Treatment with Physiologic Pacing. Trends Cardiovasc. Med..

[17] Keane J.F., Fyler D.C., Lock J.E. (2006). Nadas’ Pediatric Cardiology.

[18] Zeigler V.L., Gillette P.C., Zeigler V.L., Gillette P.C. (2001). Ventricular Arrhythmias. Practical Management of Pediatric Cardiac Arrhythmias.

